# Survival benefit of tamoxifen in male breast cancer: prospective cohort analysis

**DOI:** 10.1038/s41416-020-0857-z

**Published:** 2020-05-05

**Authors:** Holm Eggemann, Cosima Brucker, Michael Schrauder, Marc Thill, Felix Flock, Mattea Reinisch, Serban-Dan Costa, Atanas Ignatov

**Affiliations:** 1 Department of Gynaecology and Obstetrics, Otto-von-Guericke University, Magdeburg, Germany; 2 Department of Gynecology and Obstetrics, Klinikum Magdeburg, Magdeburg, Germany; 3 Breast Unit, Klinikum Nürnberg, Nürnberg, Germany; 4 Breast Unit, Klinikum Aschaffenburg, Aschaffenburg, Germany; 5 Breast Unit, Agaplesion Markus Hospital, Frankfurt, Germany; 6 Brast Unit, Brustzentrum/Klinikum Memmingen, Memmingen, Germany; 7 Breast Unit, Kliniken Essen-Mitte, Essen, Germany; 8 Department of Gynecology and Obstetrics, University Medical Center Regensburg, Regensburg, Germany

**Keywords:** Outcomes research, Adaptive clinical trial

## Abstract

**Background:**

Due to the lack of prospective data, current treatment of male breast cancer (MBC) is based on information obtained from retrospective analysis or by extrapolation from studies on female patients. In this prospectively enrolled cohort study, we retrospectively examined the survival effect of tamoxifen in MBC patients.

**Methods:**

In this prospectively enrolled cohort study, 448 patients with MBC were treated between May 2009 and June 2018. The primary endpoint was disease-free survival (DFS).

**Results:**

Between May 2009 and June 2018, 448 men with breast cancer were identified, with a median age at diagnosis of 69 years (range 27–96 years). The median follow-up was 39 months (range 3–89 months). Most tumours were larger than 20 mm; invasive ductal carcinoma was of no special histological type and with an intermediate grade of differentiation. Almost half of the men were diagnosed with positive axillary lymph nodes (43.5%). Hormone receptor (HR) positivity was observed in 98.4% of the patients. Notably, DFS among men who did not receive tamoxifen was significantly reduced as compared with those who underwent tamoxifen therapy (*P* = 0.002). The recurrence rate and mortality in the group of patients without and with tamoxifen treatment were 18.2% and 11.2%, respectively. The most common localisation of metastases was the bone. After adjustment for prognostic factors, we found that tamoxifen was found to reduce the recurrence rate by 68% (hazard ratio HR = 0.32; 95% confidence interval, CI: 0.14–0.74).

**Conclusions:**

Tamoxifen treatment was associated with improved DFS for MBC patients.

**Clinical trial registration:**

DRKS00009536.

## Background

Male breast cancer (MBC) is an uncommon disease, which likely explains the lack of prospective trials. Current treatment guidelines are based on limited retrospective studies and clinical management of female breast cancer (FBC).^1,2^ Based on the different physiology of males and females, caution should be exercised when simply extrapolating data from the management of FBC and applying it to MBC patients. Patients with MBC appear to be hormone receptor (HR)-positive in >90% of cases, and endocrine therapy remains one important treatment strategy.^2–4^ In a recent retrospective study with 257 MBC patients, we showed that tamoxifen was associated with a 1.4-fold decreased risk of cancer mortality compared with aromatase inhibitor (AI) treatment.^3^ Furthermore, tamoxifen treatment was associated with a similar overall survival (OS) for both MBC and FBC. By contrast, AI treatment is associated with poorer survival of MBC patients.^5^

In this large prospective cohort study of 448 MBC patients to be HR-positive, we investigated retrospectively the benefit of tamoxifen treatment on disease-free survival (DFS).

## Methods

We investigated cases of MBC included in the German national prospective cancer registry. This study was registered at the Deutsches Register Klinischer Studien (DRKS; DRKS00009536).^6^ The cancer registry contained the following information about MBC patients: date of diagnosis, patient and tumour characteristics, operative, neoadjuvant and/or adjuvant treatment, date and localisation of relapse, date and cause of death, secondary cancer and comorbidities. We analysed 448 men with primary breast cancer who had been diagnosed between May 2009 and July 2017. For survival analysis, we included only patients with non-metastatic invasive HR-positive breast cancer, with a minimum follow-up of 6 months.

**Fig. 1:**
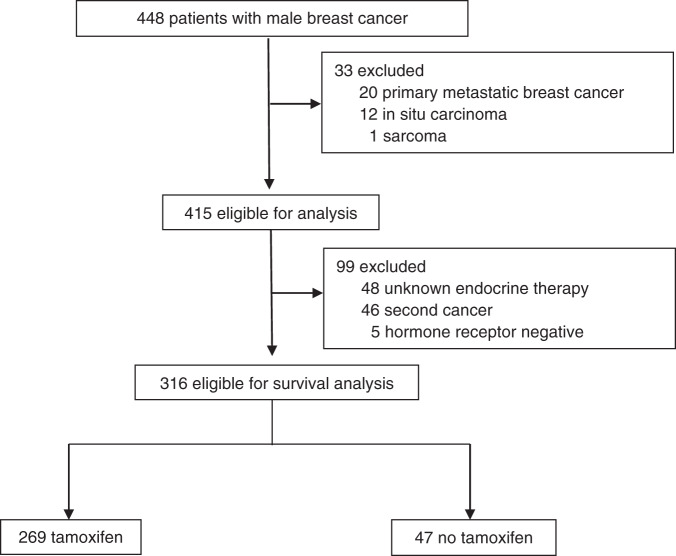
Study design.

Patients were excluded if they had primary metastatic disease (*n* = 20), in situ carcinoma (*n* = 12) or sarcoma (*n* = 1, Fig. 1). As a result, 415 patients were eligible for analysis of clinical and pathological characteristics of MBC. Among these men, we further selected only HR-positive patients and known endocrine therapy. Patients were excluded from survival analysis if they had unspecified endocrine therapy (*n* = 48), a secondary cancer (*n* = 46) and a negative HR status (*n* = 6). Thus, 316 men were eligible for survival analysis, of whom 269 underwent tamoxifen treatment and 47 had another endocrine therapy or neither endocrine or tamoxifen therapy. The grading of the tumours was assessed using Nottingham scoring system. The HR positivity was defined using a cut-off of ≥1% of receptor expression.

The primary outcome of the study was the rate of DFS, which was defined as the interval between the date of diagnosis and loco-regional recurrence, distant metastases or death from breast cancer, whichever occurred first. The study was undertaken in accordance with the Declaration of Helsinki and the guidelines for Good Clinical Practice, and was approved by the Research and Ethical Committee of Otto-von-Guericke University, Magdeburg, Germany. Before treatment, the patients gave written informed consent. The paper was prepared in accordance with the STROBE statement criteria.^7^

### Statistical analysis

Statistical analyses were performed using SPSS Version 23.0 (IBM Corp., Armonk, NY, USA). The differences between ordinal and continuous variables were assessed by chi-square and Fisher’s exact test. The DFS probability distribution was examined using the Kaplan–Meier method. For patients who remained alive and disease free or died by a cause other than breast cancer, the data were censored at the date of the last follow-up. The equality of survival curves was tested using the log-rank test. A Cox proportional hazard model was used to adjust for known clinical and pathological prognostic variables. All tests were two-sided, and the statistical significance was established if the *P* value was ≤0.05. All confidence intervals (CI) were at the 95% level.

## Results

Between May 2009 and June 2018, 448 men with breast cancer were identified, whereby 33 were excluded from the study (Fig. 1). The median follow-up was 39 months (range 2–89 months). Patient characteristics are summarised in Table 1. The median age at diagnosis was 69 years (range 27–96 years), and the median body mass index was 27.4 kg/m² (range 17–63 kg/m²). The majority of the patients had tumours larger than 20 mm (53.9%). Most tumours were invasive ductal carcinoma of no special histological type (NST, 94.1%), and most of them were of an intermediate histological grade (65.3%). Positive axillary lymph nodes were diagnosed in 43.5% of the patients. Angio-lymphatic invasion was observed in 5.8% and 40.6% of the cases, respectively. Most of the patients expressed oestrogen receptor- (ER, 97.4%) and progesterone receptor- (PR, 91.9%) positive breast cancer, whereas human epidermal growth factor receptor 2 (HER2) overexpression was observed in 14.5%. Of them, 71% received trastuzumab therapy. All patients eligible for analysis underwent an operative treatment; thereby mastectomy (95.9%) was the most common procedure. Axillary node dissection, radiotherapy and chemotherapy were undertaken in 44.8%, 51.9% and 41.0% of the cases, respectively.

**Table 1:** Clinical and pathological characteristics.

Parameter	No TAM		TAM		*P* value
	*N*	%	*N*	%	
Total	56	15.3	309	84.7	
Median age (years)	71 (49–87)		69 (27–96)		0.197
*Tumour size*					
≤20 mm	21	42.0	132	46.6	0.544
>20 mm	29	58.0	151	53.4	
Missing	6		26		
*Histological type*					0.701
Invasive ductal carcinoma of no special type	51	92.7	286	94.1	
Other	4	7.3	18	5.9	
Missing	1		5		
*Lymph node status*					
Negative	32	59.3	169	56.7	0.728
Positive	22	40.7	129	43.3	
Missing	2		11		
*Histological grade*					
1	5	8.9	32	10.5	0.907
2	38	67.9	198	65.1	
3	13	23.2	74	24.3	
Missing	0		16		
*Lymph vessel involvement*					
Negative	28	54.9	170	60.1	0.489
Positive	23	45.1	113	39.9	
Missing	5		26		
*Blood vessel involvement*					
Negative	47	94.0	263	94.6	0.863
Positive	3	6.0	15	5.4	
Missing	6		31		
*ER status*					
Negative	3	5.4	4	1.3	0.077
Positive	53	94.6	304	98.7	
Missing	0		1		
*PR status*					
Negative	4	7.1	20	6.5	0.077
Positive	52	92.9	288	93.5	
Missing	0		1		
*HER2 status*					
Negative	41	91.1	228	84.8	0.359
Positive	4	8.9	41	15.2	
Missing	11		40		

After exclusion of patients with negative HR status, unknown endocrine therapy and secondary cancer, 316 men with HR-positive breast cancer remained eligible for survival analysis (Fig. 1). These patients were divided into two groups: 269 (85.1%) men were treated with tamoxifen, and 47 (14.9%) did not receive any tamoxifen. The baseline characteristics are equally distributed between the two treatment groups (Table 1).

**Fig. 2:**
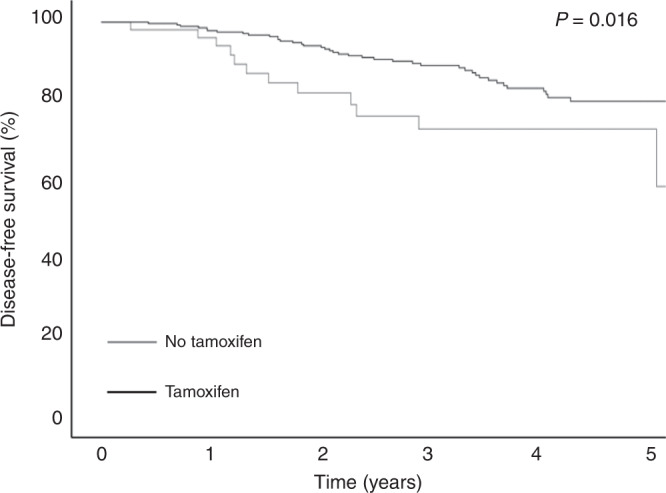
Disease-free survival. Kaplan-Meier analysis of disease-free survival depend on endocrine treatemnt.

DFS was compared between these groups. In the group of men who did not receive tamoxifen, DFS was significantly lower compared with the tamoxifen-treated group (*P* = 0.002, Fig. 2). The rate of recurrence or death in both groups without and with tamoxifen treatment was 22.6% and 13.9%, respectively. The most common site of recurrence was the bone, followed by loco-regional sites, liver, lungs and/or pleura, distant lymph nodes, brain and rectum (Table 2).

**Table 2:** Patterns of recurrence.

Site of reccurrence	*N*	%
Loco-regional	14	25.9
Bone	19	35.2
Lung/pleura	8	14.8
Liver	6	11.1
Distant nodal	4	7.4
Brain	1	1.9
Other	2	3.7

The univariate analysis demonstrated that tamoxifen treatment, tumours with good and intermediate grade of differentiation and negative lymph nodes were associated with favourable prognosis (Table 3). After adjustment for age at diagnosis, tumour size, histological type and grade, lymph node involvement and adjuvant treatment, tamoxifen represented a favourable prognostic factor in regard to DFS (hazard ratio 0.38; 95% CI: 0.19–0.78, Table 3). Undifferentiated grading and positive lymph nodes were further associated with an increased risk of recurrence with a HR of 2.61 (95% CI: 1.34–5.05) and 2.87 (95% CI: 1.42–5.76), respectively. The remaining factors did not significantly influence DFS of patients with MBC.

**Table 3:** Uni- and multivariate analysis regarding disease-free survival.

	Univariate		Multivariate	
Variable	Hazard ratio for events (95% CI)	*P* value	Hazard ratio for events (95% CI)	*P* value
*Tamoxifen*				
No	1.00	0.019	1.00	0.008
Yes	0.46 (0.24–0.88)		0.38 (0.19–0.78)	
*Age at diagnosis (yr)*				
≤69	1.00	0.227	1.00	0.274
>69	1.39 (0.82–2.37)		2.44 (0.75–2.77)	
*Tumour size*				
≤20 mm	1.00	0.165	1.00	0.327
>20 mm	1.49 (0.85–2.62)		1.38 (0.73–2.60)	
*Histological grade*				
1, 2	1.00	0.001	1.00	0.005
3	2.55 (1.51–4.33)		2.61 (1.34–5.05)	
*Histological type*				
Invasive ductal carcinoma of no special type	1.00	0.619	1.00	0.388
Other	1.30 (0.47–3.59)		1.61 (0.55–4.71)	
*Lymph node status*				
Negative	1.00	0.006	1.00	0.003
Positive	2.19 (1.25–3.83)		2.87 (1.42–5.76)	
*Chemotherapy*				
No	1.00	0.727	1.00	0.729
Yes	1.10 (0.65–1.86)		0.88 (0.42–1.83)	
*Radiation*				
No	1.00	0.668	1.00	0.205
Yes	1.13 (0.66–1.93)		0.65 (0.34–1.26)	

## Discussion

Our prospective cohort study showed that tamoxifen treatment improved DFS of patients with MBC. This study is the first prospective cohort study showing the benefit of tamoxifen treatment in MBC. These data are consistent with our recent large retrospective study,^3^ and various other retrospective studies with smaller cohorts.^8,9^ Importantly, we recently found out that the survival effect of tamoxifen treatment was comparable in both MBC and FBC.^5^ In line with the observations in this study, using matching analysis between patients with FBC and MBC, we show that AI treatment of MBC is associated with significantly decreased survival.^5^ Clearly, it might not always be appropriate to explore treatment strategies for MBC from FBC treatment guidelines, due to gender-specific differences in physiology. In men, 80% of the oestrogen is produced by aromatase, and 20% directly in the testis, which might be the reason for the ineffective suppression of oestrogen levels by AIs.^10^ Increased levels of follicle-stimulating hormone (FSH) and testosterone after AI treatment, with increased aromatisation, might be another possible explanation.^11,12^

Our study has several important strengths: (i) this is the first prospective study evaluating the effect of tamoxifen in patients with MBC, (ii) it is the largest study of adjuvant hormonal therapy in MBC patients and (iii) the exclusion criteria were kept to a minimum, providing a high external validity. It should be noted that the size of the MBC patients’ cohort without tamoxifen treatment was rather moderate. Thus, based on our data collected from an MBC cohort with a median follow-up of 39 months (range 3–89 months), we propose utilising tamoxifen as an adjuvant treatment of choice for initial treatment of HR-positive MBC.

Whether prolonged tamoxifen treatment would be beneficial for a group of patients at high risk of recurrence should be investigated in future studies, which preferably cover extended periods, e.g. 10 years. We will also use the present prospective cohort study to investigate whether prolonged tamoxifen treatment is indicated for MBC patients at high risk. Tamoxifen treatment is associated with various adverse effects such as gastrointestinal and cardiovascular problems, psychiatric disorders, erectile dysfunction, gynaecomastia, fatigue, musculoskeletal and connective tissue disorders and thromboembolic events.^13^ Specific disorders, such as gynaecomastia and erectile dysfunction, have been described in men, whereas endometrial cancer and increased rate of fractures have been observed in women.^13,14^ Recently, we reported that tamoxifen treatment in men is associated with significantly increased risk of thrombotic events in comparison with tamoxifen treatment for female breast cancer patients.^6^ All these data should be considered during treatment decision.

HR positivity is the basis for successful tamoxifen therapy in MBC. Data from studies comparing FBC and MBC suggest that MBC patients are largely HR-positive, and the rates of ER and PR cancers vary between 70 and 90%, respectively.^1,15^ In this study, 98.4% of the tumours were HR-positive. ER- and PR positivity were observed in 97.4% and 91.9% of the patients, respectively. These data were consistent with other reports showing a HR expression of more than 90% for patients with MBC.^2,3,5^

Tamoxifen treatment significantly reduces the recurrence rate in our cohort. The total encompassing recurrence and cancer-specific death in the current study is 12.5%, and thus lower than the recurrence rate observed previously.^14^ This difference might be due to their longer follow-up period of 48 months, whereas the median follow-up for our cohort was 39 months. Notably, most recurrences are observed in bone, at loco-regional sites, liver and lungs. This recurrence pattern is similar to that observed for FBC cohorts,^15^ and in other studies with MBC patients.^10^

Interestingly, in our prospective cohort analysis, we detected somehow higher HER2 expression than that previously reported. This difference might be due to the following reasons: (i) recent updates of the original ASCO/CAP guidelines, resulting in changes in the number of positive cases,^16^ (ii) different scoring systems and cut-off values used in the studies regarding HER2 expression in MBC and (iii) differences that may arise between prospective and retrospective studies. It should be noted that recent studies show an HER2 overexpression of about 15%, which is similar to our observation.^16^

## Data Availability

The raw data are available by request.

## References

[1] Losurdo A, Rota S, Gullo G, Masci G, Torrisi R, Bottai G (2017). Controversies in clinicopathological characteristics and treatment strategies of male breast cancer: a review of the literature. Crit. Rev. Oncol. Hematol..

[2] Leon-Ferre RA, Giridhar KV, Hieken TJ, Mutter RW, Couch FJ, Jimenez RE (2018). A contemporary review of male breast cancer: current evidence and unanswered questions. Cancer Metastasis.

[3] Eggemann H, Ignatov A, Smith BJ, Altmann U, von Minckwitz G, Rohl FW (2013). Adjuvant therapy with tamoxifen compared to aromatase inhibitors for 257 male breast cancer patients. Breast Cancer Res. Treat..

[4] Streng M, Ignatov A, Reinisch M, Costa SD, Eggemann H (2018). A comparison of tumour size measurements with palpation, ultrasound and mammography in male breast cancer: first results of the prospective register study. J. Cancer Res. Clin. Oncol..

[5] Eggemann H, Altmann U, Costa SD, Ignatov A (2017). Survival benefit of tamoxifen and aromatase inhibitor in male and female breast cancer. J. Cancer Res. Clin. Oncol..

[6] Eggemann H, Bernreiter AL, Reinisch M, Loibl S, Taran FA, Costa SD (2019). Tamoxifen treatment for male breast cancer and risk of thromboembolism: prospective cohort analysis. Br. J. Cancer.

[7] von Elm E, Altman DG, Egger M, Pocock SJ, Gotzsche PC, Vandenbroucke JP (2007). The strengthening the reporting of observational studies in epidemiology (STROBE) statement: guidelines for reporting observational studies. Lancet.

[8] Goss PE, Reid C, Pintilie M, Lim R, Miller N (1999). Male breast carcinoma: a review of 229 patients who presented to the Princess Margaret Hospital during 40 years: 1955-1996. Cancer.

[9] Ribeiro G, Swindell R (1992). Adjuvant tamoxifen for male breast cancer (MBC). Br. J. Cancer.

[10] Volm MD (2003). Male breast cancer. Curr. Treat. Options Oncol..

[11] Roselli CE, Resko JA (1997). Sex differences in androgen-regulated expression of cytochrome P450 aromatase in the rat brain. J. Steroid Biochem. Mol. Biol..

[12] Shetty G, Krishnamurthy H, Krishnamurthy HN, Bhatnagar AS, Moudgal NR (1998). Effect of long-term treatment with aromatase inhibitor on testicular function of adult male bonnet monkeys (*M. radiata*). Steroids.

[13] Anderson WF, Althuis MD, Brinton LA, Devesa SS (2004). Is male breast cancer similar or different than female breast cancer?. Breast Cancer Res. Treat..

[14] Henriques Abreu M, Henriques Abreu P, Afonso N, Pereira D, Henrique R, Lopes C (2016). Patterns of recurrence and treatment in male breast cancer: a clue to prognosis?. Int. J. Cancer.

[15] Ignatov A, Eggemann H, Burger E, Ignatov T (2018). Patterns of breast cancer relapse in accordance to biological subtype. J. Cancer Res. Clin. Oncol..

[16] Ottini L, Capalbo C, Rizzolo P, Silvestri V, Bronte G, Rizzo S (2010). HER2-positive male breast cancer: an update. Breast Cancer.

